# Expression of concern: Evaluation of post-COVID mortality risk in cases classified as severe acute respiratory syndrome in Brazil: a longitudinal study for medium and long term

**DOI:** 10.3389/fmed.2025.1561115

**Published:** 2025-01-15

**Authors:** Frontiers Editorial Office

With this notice, Frontiers states its awareness of concerns regarding the content of the article “**Evaluation of post-COVID mortality risk in cases classified as severe acute respiratory syndrome in Brazil: a longitudinal study for medium and long term**” published on 18th December 2024. Our Research Integrity team will conduct an investigation in full accordance with our procedures. The situation will be updated as soon as the investigation is complete. UPDATE: This article has now been retracted. Please find the full retraction here: https://doi.org/10.3389/fmed.2025.1619451.

